# Organizational communication satisfaction as a moderator between the big five personality traits and job satisfaction among employees in Nigeria

**DOI:** 10.3389/fpsyg.2024.1339305

**Published:** 2025-07-07

**Authors:** Yosra Jarrar, Lujain Ammar, Gabriel Nweke, Ibrahim Horoub, Adebola Aderibigbe

**Affiliations:** ^1^Communication and Information Studies Department, American University, Dubai, United Arab Emirates; ^2^Psychology Department, Girne American University, Kyrenia, Cyprus; ^3^Media and Communication Development - Arab American University, Ramallah, Palestine; ^4^Communication & Media Studies, Bowen University, Iwo, Nigeria

**Keywords:** job satisfaction, openness, conscientiousness, extroversion, agreeableness, neuroticism, organizational communication satisfaction

## Abstract

This study seeks to understand the relationship between job satisfaction and the Big Five personality traits—openness, conscientiousness, extroversion, agreeableness, and neuroticism—while considering the moderating effect of organizational communication satisfaction. Given the recent emphasis on evaluating employee traits in the workplace, both in positive and negative contexts, this research aims to delve deeper into how these major personality traits influence and potentially enhance workplace dynamics, particularly during periods of fluctuating wellbeing. Using convenience sampling, the study focuses on employees at Bowen University in Nigeria to explore the relationship between job satisfaction and the Big Five personality traits, with organizational communication satisfaction serving as the moderating variable. The independent variables include the five personality traits, while job satisfaction serves as the dependent variable. The findings revealed a positive relationship between job satisfaction and the personality traits of openness, conscientiousness, extroversion, and agreeableness, whereas a negative relationship was observed with neuroticism. Additionally, a positive correlation was found between organizational communication satisfaction and job satisfaction. As a moderator, organizational communication satisfaction played a positive role in moderating the relationship between openness, extroversion, agreeableness, and neuroticism with job satisfaction. However, this moderating effect was not supported in the case of openness and job satisfaction, as well as conscientiousness and job satisfaction. Overall, the study confirms the significant impact of the Big Five personality traits on job satisfaction, particularly in relation to organizational communication satisfaction.

## Introduction

Navigating the modern work landscape across various fields presents challenges, especially when viewed through the lens of major historical economic phenomena such as the Great Depression and the Industrial Revolution. In response, the study of employee wellbeing, particularly through the lens of job satisfaction, has emerged (Judge et al., 2020). Job satisfaction, a central concept in organizational studies and behavior, has become increasingly pivotal for analysis in workplaces, especially during times of crisis. Its correlations extend to various aspects, such as task performance, employee turnover, absenteeism, organizational citizen behavior (OCB), and counterproductive work behavior, each characterized by its unique definition (Judge et al., 2020). At its core, job satisfaction reflects an individual's perception and evaluation of their job, influenced by circumstances including needs, values, and expectations (Buitendach and De Witte, 2005).

The exploration of job satisfaction spans numerous dimensions, including social relationships, organizational culture, peer dynamics, and additional variables that externalize its manifestation and impact on emotions and feelings. However, a comprehensive understanding of job satisfaction, rooted in internal observations, necessitates an examination of its relationship with the fundamental Big Five personality traits. These traits encompass a spectrum of emotions, habits, and attitudes that form the foundational characteristics of individuals in any environment, particularly in the workplace. Initially conceptualized by D.W. Fiske in 1949, scholars have refined and expanded upon these traits through decades of behavioral and theoretical discourse (Jingli, 2018). The breakdown of these traits begins with extroversion, which encompasses attributes such as being active, talkative, sociable, and assertive—qualities crucial in a workplace setting. Furthermore, extroversion encompasses motivation, ambition, and the ability to adapt creatively to external relationships and surroundings (Chiang et al., 2017; Barrick and Mount, 1991). Next is agreeableness, characterized by kindness, consideration, and warmth in social interactions (Graziano and Tobin, 2009). This trait emphasizes selflessness and prioritizing the needs of others, which is essential for effective collaboration with diverse personalities. Conscientiousness, the third trait, entails a focused approach, diligence, and a planned mindset—qualities vital for goal-directed behavior and adeptness in handling various situations (Roberts et al., 2009). Openness follows as the intellectual aspect, associated with imagination, curiosity, innovation, and originality—essential for fostering creativity and thinking beyond conventional norms (DeYoung et al., 2014). Finally, neuroticism, the fifth trait, embodies emotional instability, anxiety, self-consciousness, and depression—a trait whose presence in the workplace should be minimized due to its adverse effects (Widiger and Oltmanns, 2017).

While the Big Five personality traits have been studied in work environments, their significance concerning job satisfaction remains understudied. Previous research has explored their role in person-job fit environments and their impact on job satisfaction (Ghetta et al., 2020). However, understanding the nuanced influence of each Big Five trait on job satisfaction necessitates further investigation. Isolating the influence of these traits on job satisfaction alone overlooks the need for a moderator variable that can effectively contribute to both variables. Organizational communication satisfaction emerges as a crucial moderator in this context, facilitating a deeper understanding of the interplay between the Big Five personality traits and job satisfaction.

Organizational communication satisfaction comprises two essential components: organizational culture/format and communication satisfaction. Once an organization establishes its work culture and guidelines, effective communication becomes a primary objective, facilitating the clear exchange of information among employees (Sharma, 2015). Numerous research papers have highlighted the importance of organizational communication satisfaction for various factors, including employee engagement, job satisfaction, and job involvement (Iyer and Israel, 2012). Conversely, insufficient organizational communication satisfaction can breed distrust, increase employee turnover, foster uncertainty among employees, and contribute to overall workplace dissatisfaction—factors that can hinder the achievements of the company or team (Gill and Sypher, 2010; Earley, 1986). In the context of workplace dynamics, organizational communication satisfaction is predicted to significantly influence employee behavior and attitudes, particularly as a moderator in this study, where it interacts with the Big Five personality traits and their relationship with job satisfaction. Considering these variables, the study is situated within an educational setting that necessitates extensive communication across various structures—from students to peers to academics. Educational facilities exemplify the importance of job satisfaction through effective communication in diverse formats. Thus, examining whether organizational communication satisfaction, as a moderating variable, can effectively interact with the Big Five personality traits and their potential influence on job satisfaction becomes crucial.

### Job characteristics model

When examining the influence of the Big Five personality traits on job satisfaction, with organizational communication satisfaction as a moderator, the study's theoretical underpinning traces back to the Job Characteristics Model (JCM). Originating in 1976 by Richard Hackman and Greg Oldham, the JCM is renowned for its adaptability to various job-design formats and efforts aimed at enhancing workplace conditions (Faraji et al., 2008). The model seeks to identify the attributes and factors that contribute to an ideal workplace across satisfaction, performance, and other dimensions. In a study conducted by Raihan (2020), the Job Characteristics Model was explored in the context of job satisfaction, particularly within organizations in Bangladesh. The study affirmed the model's core dimensions and attributes, including skill variety, task identity, task significance, autonomy, and feedback (Raihan, 2020). Skill variety pertains to the range of tasks and skills an employee handles, while task identity defines the criteria for task completion or elucidates its overarching goal. Task significance evaluates the impact of work on external parties, such as communities or individuals, while autonomy reflects the degree of freedom an employee has in executing their tasks. Lastly, feedback refers to the communication provided to employees regarding their performance (Johansson et al., 2020). These core attributes of the model have been consistently linked to employees' levels of job satisfaction, establishing a robust foundation for its relevance in this domain. Furthermore, the Job Characteristics Model has significantly contributed to understanding the relationship between job characteristics and job satisfaction. A study conducted in Tehran, Iran, specifically correlated the model with the medical field in a university setting, demonstrating its applicability across diverse contexts (Faraji et al., 2008).

Due to its expanding applications and utility, the Job Characteristics Model has been adapted and developed across various sectors, serving as the foundation for this paper. However, criticisms, particularly regarding its accuracy and relevance to the broader topic, have surfaced. Early research during its development involved analyzing 200 different studies to assess the model's validity. It was found that while the model accounts for psychological, behavioral, and personal factors and outcomes, it lacks a robust correlation with psychological states (Fried and Ferris, 1987). Moreover, because of the evolving nature of organizational structures, the model may yield inconclusive results in certain contexts. Nevertheless, the model's connection to job satisfaction and its influencing factors can be explored and clarified. This study examines the interplay between the Big Five personality traits, job satisfaction, and organizational communication satisfaction, providing a deeper understanding of how they relate to the model's overall efficacy.

### Aim

This study aims to explore the correlation between the Big Five personality traits—openness, conscientiousness, extroversion, agreeableness, and neuroticism—and job satisfaction while also considering the moderating effect of organizational communication satisfaction. Employing convenience sampling, the research focuses on employees at Bowen University in Nigeria. By examining how each trait influences job satisfaction, the study aims to ascertain their respective impacts on overall employee wellbeing.

## Hypotheses

This study posits the following hypotheses regarding the relationship between the Big Five personality traits—openness, conscientiousness, extroversion, agreeableness, and neuroticism—and job satisfaction, taking into account the moderating influence of organizational communication satisfaction.

- H1. Neuroticism will have a significant negative relationship with employee job satisfaction, while extroversion, openness, agreeableness, and conscientiousness will have a significant positive relationship with it.
- H2. Organizational communication satisfaction has a significant positive relationship with employee job satisfaction.
- H3. Organizational communication satisfaction will moderate the relationship between personality traits (e.g., extroversion, openness to experience, conscientiousness, and agreeableness) and job satisfaction. Higher levels of organizational communication satisfaction will strengthen the positive association, while the negative relationship between neuroticism and job satisfaction will be mitigated by higher levels of organizational communication satisfaction.

## Literature review

The Big Five personality traits are generally associated with positive behaviors, except for neuroticism, which encompasses traits such as anxiety and emotional instability. Consequently, neuroticism is commonly linked to negative emotions, along with reduced productivity and satisfaction at work. For instance, a study by Farfán et al. (2020) examined the roles of extroversion and neuroticism as moderators in the relationship between work autonomy, burnout, and job satisfaction. The findings revealed that extroversion was positively correlated with job satisfaction and work autonomy but negatively correlated with emotional exhaustion. Conversely, neuroticism showed a positive relationship with emotional exhaustion while demonstrating a negative relationship with job satisfaction and work autonomy. As a moderator, neuroticism was associated with a negative relationship with job satisfaction, according to the 2020 study. However, when considering neuroticism as a broader influencing factor rather than solely a moderator, its impact on job satisfaction warrants further investigation, particularly within educational environments where various emotions intersect and when treated as a primary variable rather than a mere moderator.

Extroversion, recognized as a crucial trait, has been found to have a significant relationship with job satisfaction, as evidenced by a 2012 study conducted in China. This study examined the wellbeing of 818 employees to assess the overall impact of the Big Five personality traits on job satisfaction and subjective wellbeing (Zhai et al., 2013). The results revealed that only extroversion influenced job satisfaction, which served as a moderator between extroversion and subjective wellbeing. The study identified three traits, including extroversion, neuroticism, and conscientiousness, linked to subjective wellbeing. However, it is important to note that the study's scope was limited to China, necessitating further investigation within the Nigerian context. Moreover, by broadening the focus to include job satisfaction on a wider scale within this study, its significance in relation to extroversion, along with the moderating effect of organizational communication satisfaction, can be examined more thoroughly.

The trait of openness within the Big Five personality traits is often associated with a willingness to experience new experiences and ideas. Due to its broad relevance, its influence extends across various outcomes related to actions, behaviors, and characteristics. In a 2001 study involving 95 graduate students working part-time jobs, openness to experience, along with growth need strength (GNS), was examined as a moderator between job characteristics and job satisfaction (De Jong et al., 2001). The study revealed highly significant findings regarding the role of openness to experience. Notably, openness exhibited a stronger moderating effect than GNS concerning job skill variety. Furthermore, openness demonstrated a substantial impact on job satisfaction, comparable to that of GNS. This underscores the importance of openness in promoting positive growth within the job domain, particularly among graduate students. It suggests that openness's influence on job satisfaction goes beyond mere moderation and highlights its direct contribution to fostering satisfaction in the workplace.

Regarded as one of the most altruistic traits among the Big Five, agreeableness reflects a disposition toward prioritizing the satisfaction and wellbeing of others above one's own, fostering a propensity to aid others. In the workplace, the impact of agreeableness is multifaceted, with one notable association being its potential to enhance team performance. A study conducted in 2013 aimed to explore how individuals' agreeableness manifests in team communication, cohesion, and performance dynamics. The findings revealed that teams comprising individuals with higher levels of agreeableness demonstrated superior performance and communication in face-to-face interactions, whereas those engaging in virtual communication exhibited lower levels of both communication and performance. This underscores the nuanced ways in which agreeableness influences team collaboration through various modes of interaction (Bradley et al., 2013). However, it is noteworthy that while agreeableness may enhance team performance, it is considered one of the weakest predictors of individual job performance despite its inherent inclination to assist others. Nevertheless, when contextualized within successful team collaboration and performance, agreeableness may contribute significantly to job satisfaction, potentially surpassing the perceptions outlined in the existing literature. Moreover, an intriguing aspect worth exploring further is the interplay between agreeableness and conscientiousness concerning job performance. A 2002 study aimed to elucidate this relationship by hypothesizing that conscientiousness would have a stronger impact on job performance for individuals with high agreeableness compared to those with lower levels of agreeableness (Witt et al., 2002). While the study anticipated a correlation between agreeableness and conscientiousness, its findings predominantly underscored a robust association between high levels of conscientiousness and elevated job performance. Interestingly, within the spectrum of agreeableness, the study revealed that individuals exhibiting both high agreeableness and conscientious traits tended to demonstrate superior job performance, highlighting a positive relationship between these traits.

Organizational communication satisfaction, within its broad scope of study, has demonstrated notable connections to various concepts associated with job satisfaction, establishing it as a promising moderator in this study. A 2011 study of 150 nurses in Seoul, Korea, examined the influence of emotional intelligence—a concept similar to the Big Five personality traits—on organizational communication satisfaction and job satisfaction (Han and Lee, 2011). The findings underscored a strong correlation between emotional intelligence, organizational communication satisfaction, and job satisfaction, indicating that higher levels of job satisfaction are associated with increased emotional intelligence and organizational communication satisfaction. This suggests a link between organizational communication and job satisfaction, thereby positioning organizational communication satisfaction as a moderator, considering its relationship with behavior and job satisfaction factors. However, the moderating role of organizational communication satisfaction goes beyond a single variable and deserves further investigation within the context of the Big Five personality traits. For instance, a 2006 study investigated the impact of communication satisfaction on job satisfaction while accounting for individual differences in the Big Five personality traits in Taiwan. The findings revealed that extroversion, openness to experience, agreeableness, and conscientiousness positively predicted both communication satisfaction and job satisfaction, whereas neuroticism showed no significant relation to job satisfaction, indicating an inverse relationship. Although both aspects were connected, the study did not explicitly acknowledge the role of organizational communication satisfaction as a moderator, a gap that this study aims to address. Similarly, a 2014 study assessed the correlation between communication satisfaction and job satisfaction in Mashhad, Iran, through the lens of the Big Five personality traits—nervousness, extroversion, empiricism, responsibility, and compatibility (Arabshahi and Arabshahi, 2014). While the findings revealed weak to no significant relationships between extroversion, empiricism, responsibility, and compatibility, they indicated a negative relationship between nervousness and job satisfaction. Moreover, a strong correlation emerged between organizational communication satisfaction and job satisfaction. Despite yielding weak results, the study substantiates the existence of a relationship between communication satisfaction and personality traits, underscoring the need for further analysis when considering organizational communication satisfaction as a significant moderator.

In reviewing the existing literature concerning the variables under examination, this study aims to delve into and scrutinize each aspect of personality traits, job satisfaction, and organizational communication satisfaction. By exploring the proposed hypotheses, the goal is to chart a novel course toward enhancing employees' overall wellbeing. This contribution fosters a deeper understanding of the workplace environment within an educational facility, where various traits and personalities are observed and interact. Through this exploration, the study seeks to illuminate pathways for optimizing employee welfare and improving organizational dynamics.

## Methodology

### Research design

This study utilized an ex-post facto correlational research design of a descriptive type employing a non-experimental research approach. According to Kerlinger et al. (2000), this methodology entails a systematic empirical inquiry where the researcher has no direct control over the independent variable, as its manifestations have already transpired. In this study, the independent variables encompass personality traits, specifically openness, conscientiousness, extraversion, agreeableness, and neuroticism, while job satisfaction serves as the dependent variable. Organizational communication satisfaction is identified as the moderator variable, influencing the relationship between the independent and dependent variables.

### Population and sampling

To investigate the moderating influence of organizational communication satisfaction on the relationship between personality and job satisfaction, employees from Bowen University in Nigeria were selected as the target population for this study. Data collection utilized a convenience sampling technique, gathering responses from both academic and non-academic staff members of the university. Self-report and closed-ended questionnaires were employed for data collection. Using the Slovin method for sample size determination, a minimum of 342 participants was required to ensure the study's external validity. A total of 500 questionnaires were manually distributed among participants, yielding 445 returned surveys, resulting in an 89% response rate. Some responses were omitted due to non-response or incomplete surveys, reducing the final number of valid responses to 438 for analysis. All research ethical principles were strictly adhered to, and participation in the study was voluntary. Demographically, the respondents comprised 62.3% men and 38.7% women, with an average age of 51 years.

### Measures

#### Personality

The Big Five Inventory (BFI) served as the instrument for gathering data on personality traits. Developed by John, Donahue, and Kentle in 1991, the BFI comprises 44 items designed to measure the five personality dimensions: Extroversion, Agreeableness, Conscientiousness, Neuroticism, and Openness. Example items include “Is talkative” for Extroversion, “Is helpful and unselfish with others” for Agreeableness, “Does a thorough job” for Conscientiousness, “Feels depressed or blue” for Neuroticism, and “Is curious about many different things” for Openness. Participants rated each BFI item on a 5-point scale ranging from 1 (strongly disagree) to 5 (strongly agree), with scale scores calculated as the mean of item responses. Cronbach's alpha was employed to assess the reliability of the scale. The reliability coefficient ranged from 0.78 to 0.88 for all five personality dimensions, indicating satisfactory internal consistency and reliability of the BFI in measuring these traits.

#### Job satisfaction

Employee job satisfaction was assessed using the Job Satisfaction Scale (JSS) developed by Dubey et al. (1983). This scale comprises 25 items designed to gauge employees' satisfaction levels in the workplace. Example items include “I find I have to work harder at my job because of the incompetence of people I work with” and “The benefits we receive are as good as most other organizations offer.” The reliability of the scale was evaluated using Cronbach's alpha, yielding a coefficient of 0.84. This indicates a high level of internal consistency and reliability of the JSS in measuring job satisfaction among employees.

#### Organizational communication satisfaction

Organizational communication satisfaction was assessed using a 25-item scale adapted from the Communication Satisfaction Questionnaire (CSQ) developed by Downs and Hazen (1977). This scale utilizes a 7-point rating format ranging from “very dissatisfied” to “very satisfied.” Sample items included “information about my progress.” The reliability of this scale was determined to be 0.794, indicating a satisfactory level of internal consistency and reliability in measuring organizational communication satisfaction among participants.

### Data analysis

The data collected were analyzed using SPSS 24.0 for Windows and JAMOVI. In line with the research objective, Pearson's correlation analysis was conducted to examine relationships between variables, and moderation analysis was conducted to analyze interaction effects.

## Presentation of findings

Tables 1, 2 present the descriptive statistics and correlation matrix for all variables. The findings reveal a significant negative correlation between neuroticism and job satisfaction among participants. Additionally, the results demonstrate positive relationships between extroversion, agreeableness, conscientiousness, openness, and job satisfaction. Moreover, organizational communication satisfaction exhibits a positive association with job satisfaction, indicating that a higher level of organizational communication satisfaction is linked to greater job satisfaction.

**Table 1:** Descriptive.

				**Shapiro-Wilk**			
	**Mean**	**SD**	**Cronbach**α	**Skewness**	**Kurtosis**	***W***	***p***
Job satisfaction	3.45	0.721	0.840	0.466	1.191	0.951	0.101
Agreeableness	2.50	0.450	0.798	163	1.017	0.957	0.211
conscientiousness	2.18	0.581	0.880	−0.318	−0.182	0.970	0.184
Extroversion	2.73	1.05	0.815	0.442	0.999	0.956	0.521
Openness	3.18	0.788	0.838	−0.251	−0.294	0.987	0.362
Neuroticism	3.40	1.03	0.780	−0.573	−0.302	0.984	0.241
OCS	4.07	1.09	0.901	1.016	0.898	0.975	0.191

OCS, organizational communication Satisfaction.

**Table 2:** Correlation matrix.

	**Con**	**Agreeable**	**Openness**	**Extroversion**	**Neuroticism**	**OCC**	**JS**
Con							
Agreeable	0.165^*^						
Openness	0.156^**^	0.122^*^					
Extroversion	0.235^***^	0.076	0.541^**^				
Neuroticism	0.104^*^	−0.168^**^	−0.092^*^	0.085			
OCS	0.276^***^	0.112^*^	0.46^**^	0.728^***^	0.069		
JS	0.178^*^	0.416^**^	0.292^**^	0.343^***^	−0.133^**^	0.287^***^	—

^*^p < 0.05, ^**^p < 0.01, ^***^p < 0.001. OCS, organizational communication satisfaction; JS, Job Satisfaction.

**Figure 1:**
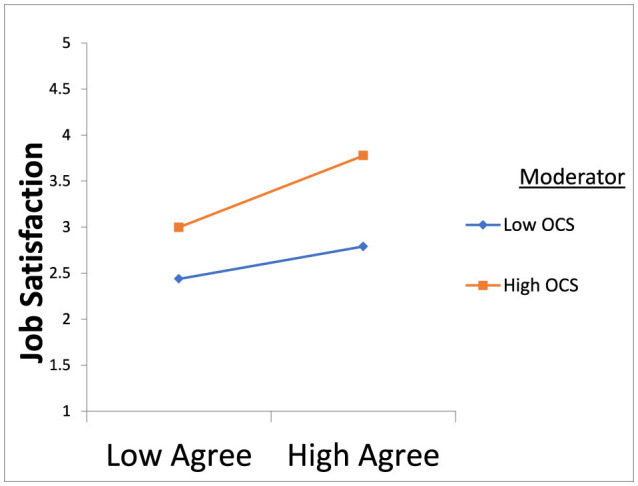
OCS reduces the strength of the relationship between Neuroticism and job satisfaction.

**Figure 2:**
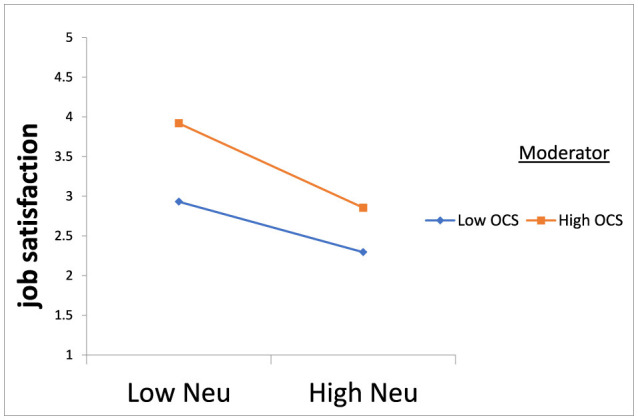
OCS strengthens the positive relationship between extroversion and job satisfaction.

**Figure 3:**
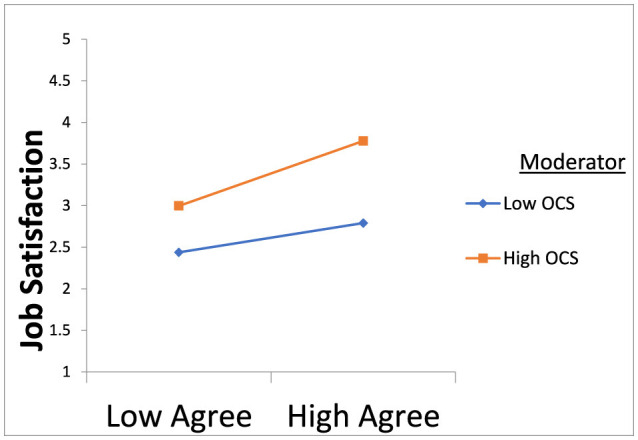
OCS strengthens the positive relationship between agreeableness and job satisfaction.

The MedMod technique in JAMOVI 1.8.4.0 software was utilized to examine the moderation hypotheses. A moderation test was conducted, with neuroticism, agreeableness, extroversion, conscientiousness, and openness serving as predictors, job satisfaction as the dependent variable, and organizational communication satisfaction as the moderator. The results showed in Tables 3, 4 indicate that organizational communication satisfaction significantly moderates the relationship between neuroticism and job satisfaction as shown in Figure 1. Specifically, participants who reported higher-than-average levels of organizational communication satisfaction experienced a more pronounced impact of neuroticism on job satisfaction compared to those with average or lower-than-average levels of organizational communication satisfaction. Moreover, organizational communication satisfaction moderates the relationship between extroversion and job satisfaction, supporting the hypothesis as shown in Figure 2. Participants with average levels of organizational communication satisfaction experienced a stronger positive effect of extroversion on job satisfaction compared to those with higher or lower-than-average levels of organizational communication satisfaction. Similarly, organizational communication satisfaction moderates the relationship between agreeableness and job satisfaction as shown in Figure 3. Participants with average levels of organizational communication satisfaction experienced a stronger positive effect of agreeableness on job satisfaction compared to those with higher or lower-than-average levels of organizational communication satisfaction.

**Table 3:** Moderation analysis.

**Predictors**	**Job satisfaction**					
	**B**	**SE**	**95% CI**		**z**	**p**
			**Lower**	**Upper**		
Neuroticism	−0.4256	0.0453	−0.51470	−0.3365	−9.391	<0.001
Openness	0.0475	0.0268	−0.00526	0.1002	1.770	0.078
Conscientiousness	0.0229	0.0295	−0.03510	0.0809	0.776	0.438
Extroversion	0.4587	0.0429	0.37430	0.5431	10.681	<0.001
Agreeableness	0.2829	0.0317	0.22070	0.3452	8.934	<0.001
OCS	0.3867	0.0526	0.28335	0.4900	7.356	<0.001
**Moderation**					
Neuroticism × OCS	−0.107	0.013	−0.103	−0.081	2.01	0.041
Openness × OCS	−0.001	0.003	−0.008	0.005	−0.352	0.725
Conscientiousness × OCS	0.003	0.003	−0.005	0.009	0.592	0.554
Extroversion × OCS	0.109	0.041	0.0545	0.179	2.12	0.034
Agreeableness × OCS	0.107	0.031	0.102	0.126	1.99	0.042

**Table 4:** Simple slope estimates.

			**95% Confidence Interval**	
	**Estimate**	**SE**	**Lower**	**Upper**	**t**	**p**
**Neuroticism**						
Average	−0.306	0.0482	−0.400	−0.2111	−6.34	<0.001
Low (−1SD)	−0.236	0.0698	−0.373	−0.0996	−3.39	<0.001
High (+1SD)	−0.375	0.0463	−0.465	−0.2840	−8.10	<0.001
**Extroversion**						
Average	0.367	0.0302	0.308	0.427	12.15	<0.001
Low (−1SD)	0.303	0.0471	0.211	0.396	6.54	<0.001
High (+1SD)	0.331	0.0375	0.358	0.505	11.52	<0.001
**Agreeableness**						
Average	0.367	0.0302	0.308	0.427	12.15	<0.001
Low (−1SD)	0.431	0.0375	0.358	0.507	11.51	<0.001
High (+1SD)	0.303	0.0471	0.211	0.396	6.44	<0.001

This shows the effect of the predictor (Neuroticism, Agreeableness, and Extroversion) on the dependent variable (Job Satisfaction) at different levels of the moderator (OCS).

## Discussion

Following an examination of the Big Five personality traits and their impact on job satisfaction, particularly in light of organizational communication satisfaction as a moderator, several significant connections within the studied framework emerge. Utilizing the Job Characteristics Model alongside the examined variables—including personality traits and job satisfaction—reveals a clear understanding of how the Big Five personality traits can influence job satisfaction. This influence, both positive and negative, is consistently demonstrated and emphasized throughout this research, highlighting the profound impact of personality traits on job satisfaction and overall wellbeing.

The findings highlight a significant relationship between these main variables, indicating that through the Job Characteristics Model, the Big Five personality traits serve as essential tools in shaping the framework of job satisfaction, ultimately leading to individualized outcomes for each employee. Moreover, the study underscores the role of organizational communication satisfaction as a moderator. Although not traditionally considered a primary determinant of job satisfaction, the findings demonstrate a noteworthy relationship between organizational communication satisfaction and job satisfaction, particularly in relation to the effects of the traits. Within the scope of the Job Characteristics Model, organizational communication satisfaction emerges as a key factor in enhancing job satisfaction. Through this framework, the attributes of personality traits, organizational communication satisfaction, and job satisfaction can be further explored across various contexts and environments.

The study suggests that organizational communication satisfaction serves as an excellent tool for enhancing job satisfaction among individuals. Furthermore, it explores how this satisfaction effectively moderates the relationship between personality traits and job satisfaction. By highlighting its external influence on employees, the study sheds light on how the workplace environment and social dynamics impact employees' personality traits and overall satisfaction levels. This underscores the importance of fostering effective organizational communication practices to create a positive work environment and enhance employee satisfaction.

Upon further analysis of the hypotheses and literature reviewed, several similarities and differences emerge. Farfán et al. (2020) study discussed the role of neuroticism as a moderator in relation to job satisfaction, revealing a negative relationship. Similarly, this study also found a negative relationship with neuroticism, although it examined it as a main variable rather than a moderator. Despite this difference, both studies underscore the detrimental impact of neuroticism on job satisfaction. Extroversion, as examined in both study Farfán et al. (2020) and Zhai et al. (2013), demonstrated consistent effects on job satisfaction and subjective wellbeing. Although job satisfaction was treated as a moderator in the former study, this study found a positive relationship between extroversion and job satisfaction, highlighting their significant connection irrespective of their roles. The trait of openness to experience, analyzed in the 2001 study, showed a strong correlation with job characteristics and job satisfaction, particularly regarding job skills. This study revealed a powerful positive correlation between openness to experience and job satisfaction, emphasizing its importance in the workplace, particularly as a main variable rather than a moderator. Agreeableness, known for its cohesive properties, as demonstrated in Bradley et al. (2013) paper, exhibited a strong connection to team performance. However, this study uncovered a positive relationship between agreeableness and job satisfaction, indicating its significance on an individual scale and its potential to enhance job satisfaction, contrary to the perceptions in the 2013 paper. Conscientiousness, examined alongside agreeableness in (Witt et al., 2002) study, revealed a positive connection to job performance. In this study, conscientiousness demonstrated a similar positive relationship with job satisfaction when analyzed as an independent main variable, underscoring its importance and significant role in increasing job satisfaction.

When considering the role of organizational communication satisfaction as a moderator, a nuanced understanding emerges from the literature reviewed in comparison to this study. Han and Lee (2011) study in South Korea solidified the correlation between organizational communication satisfaction and job satisfaction, further supported by its moderator role, demonstrating that higher organizational communication satisfaction corresponds to increased levels of job satisfaction. However, the same cannot be universally said for the Big Five personality traits. In Tseng (2006) study, communication satisfaction exhibited positive relationships with extroversion, openness to experience, agreeableness, and conscientiousness but showed no relationship with neuroticism. Nonetheless, when organizational communication satisfaction was introduced as a moderator between these traits and job satisfaction, it moderated a negative relationship between neuroticism and job satisfaction. While it facilitated positive relationships between employee satisfaction extroversion and agreeableness, it did not have a similar effect on conscientiousness and openness to experience, aligning with the weak correlations found in Arabshahi and Arabshahi (2014). This discrepancy may be attributed to the personal nature of traits like openness to experience and conscientiousness, which rely less on external factors for influence. Openness to experience, for instance, is closely linked to individual intellect. Thus, while organizational communication satisfaction can significantly influence the impact of some traits on employee satisfaction, others may operate autonomously without the need for moderation. Nonetheless, they may still be interconnected on a broader scale.

## Conclusion

The Big Five personality traits, a foundational and extensively examined behavioral concept, have a significant influence in the workplace. Various studies have scrutinized their impact on work communication, performance, and satisfaction, consistently revealing their relevance across different contexts. Notably, this study highlights their role in employee satisfaction within Nigeria's educational sector. Here, neuroticism shows a positive relationship, while conscientiousness exhibits a negative one, aligning with their respective definitions. Furthermore, by introducing organizational communication satisfaction as a moderator, this research not only advances the understanding of factors contributing to job satisfaction but also explores the nuanced interplay with each personality trait. This aspect, often overlooked in previous studies, enriches the comprehension of their relationships. It is clear that organizational communication satisfaction affects certain traits differently, suggesting a need for further investigation into the mechanisms underlying these variations. Overall, this study underscores the significance of the Big Five personality traits, organizational communication satisfaction, and employee satisfaction, offering valuable insights for fostering improved communication, satisfaction, and performance cultures within the institutional realm. These findings serve as a practical guide for enhancing the overall work experience across diverse dimensions.

### Limitations and recommendations

The study's limitations are mainly due to its focused scope, which only covers a specific location and institution. Additionally, it limits its examination to workplace values, such as job satisfaction and organizational communication satisfaction, without considering a broader range of workplace attributes. Moreover, participant demographics, such as their positions, age, and educational backgrounds, are not thoroughly analyzed. Finally, the definition of job satisfaction in the study emphasizes internal behavioral aspects, potentially overlooking external factors. Recommendations: To address these limitations, future research could benefit from expanding the participant pool to include various locations and industries while also exploring a wider array of workplace values and attributes. Furthermore, a deeper exploration of the correlation between organizational communication satisfaction and individual personality traits could provide valuable insights into their combined impact on job satisfaction. Moreover, studying how these traits influence external aspects of job satisfaction, possibly at a team level, would offer a more holistic understanding of their effects.

## Data Availability

The original contributions presented in the study are included in the article/supplementary material, further inquiries can be directed to the corresponding author.
